# 11-Deoxycorticosterone Producing Adrenal Hyperplasia as a Very Unusual Cause of Endocrine Hypertension: Case Report and Systematic Review of the Literature

**DOI:** 10.3389/fendo.2022.846865

**Published:** 2022-03-31

**Authors:** Queralt Asla, Helena Sardà, Enrique Lerma, Felicia A. Hanzu, María Teresa Rodrigo, Eulàlia Urgell, José Ignacio Pérez, Susan M. Webb, Anna Aulinas

**Affiliations:** ^1^ Department of Endocrinology and Nutrition, Hospital de la Santa Creu i Sant Pau, Barcelona, Spain; ^2^ Sant Pau Biomedical Research Institute (IIB-Sant Pau), Hospital de la Santa Creu i Sant Pau, Barcelona, Spain; ^3^ Department of Medicine, University of Vic-Central University of Catalonia, Vic, Spain; ^4^ Department of Medicine, Universitat Autònoma de Barcelona, Bellaterra, Spain; ^5^ Department of Pathological Anatomy, Hospital de la Santa Creu i Sant Pau, Barcelona, Spain; ^6^ Department of Endocrinology and Nutrition, Hospital Clínic, Barcelona, Spain; ^7^ Institut d’Investigacions Biomèdiques August Pi i Sunyer, Barcelona, Spain; ^8^ Department of Medicine, Faculty of Medicine and Health Sciences, University of Barcelona, Barcelona, Spain; ^9^ Department of Pathological Anatomy, Hospital Clínic, Barcelona, Spain; ^10^ Department of Biochemistry, Hospital de la Santa Creu i Sant Pau, Barcelona, Spain; ^11^ Department of General and Digestive Surgery, Hospital de la Santa Creu i Sant Pau, Barcelona, Spain; ^12^ Centro de Investigación Biomédica en Red de Enfermedades Raras (CIBER-ER, Unit 747), Instituto de Salud Carlos III (ISCIII), Madrid, Spain

**Keywords:** mineralocorticoid hypertension, mineralocorticoid excess, 11-deoxycorticosterone (DOC), DOC-producing adrenal tumor, adrenal hyperplasia, adrenocortical carcinoma, adrenal adenoma

## Abstract

**Background and Objectives:**

11-deoxycorticosterone overproduction due to an adrenal tumor or hyperplasia is a very rare cause of mineralocorticoid-induced hypertension. The objective is to provide the most relevant clinical features that clinicians dealing with patients presenting with the hallmarks of hypertension due to 11-deoxycorticosterone-producing adrenal lesions should be aware of.

**Design and Methods:**

We report the case of a patient with an 11-deoxycorticosterone-producing adrenal lesion and provide a systematic review of all published cases (PubMed, Web of Science and EMBASE) between 1965 and 2021.

**Results:**

We identified 46 cases (including ours). Most cases (31, 67%) affected women with a mean age of 42.9 ± 15.2 years and presented with high blood pressure and hypokalemia (average of 2.68 ± 0.62 mmol/L). Median (interquartile range) time from onset of first suggestive symptoms to diagnosis was 24 (55) months. Aldosterone levels were low or in the reference range in 98% of the cases when available. 11-deoxycorticosterone levels were a median of 12.5 (18.9) times above the upper limit of the normal reference range reported in each article and overproduction of more than one hormone was seen in 31 (67%). Carcinoma was the most common histological type (21, 45.7%). Median tumor size was 61.5 (60) mm. Malignant lesions were larger, had higher 11-deoxycorticosterone levels and shorter time of evolution at diagnosis compared to benign lesions.

**Conclusions:**

11-deoxycorticosterone-producing adrenal lesions are very rare, affecting mostly middle-aged women with a primary aldosteronism-like clinical presentation and carcinoma is the most frequent histological diagnosis. Measuring 11-deoxycorticosterone levels, when low aldosterone levels or in the lower limit of the reference range are present in hypertensive patients, is advisable.

**Systematic Review Registration:**

Open Science Framework, 10.17605/OSF.IO/NR7UV.

## Introduction

Most cases of hypertension are essential or idiopathic (primary hypertension) although 5–10% of hypertensive patients present an identifiable cause (secondary hypertension) (1). Primary aldosteronism (PA) is a mineralocorticoid-induced hypertension due to an excess of aldosterone (ALD) production. PA is the most frequent endocrine cause of secondary high blood pressure (BP) (2) and the main clinical presentation includes hypertension of variable degree, but hypokalemia only in 9–37% of the cases (3). However, there are other less common non-ALD-dependent causes of mineralocorticoid hypertension with a clinical presentation similar to PA, due to overproduction of different mineralocorticoid precursors like 11-deoxycorticosterone (DOC) or other intermediate steroids (4). A small number of case reports on adrenal lesions (either adenoma, carcinoma or hyperplasia) producing mineralocorticoid intermediate metabolites have been reported, although some are probably overlooked, since intermediate measurements of steroids are not available in clinical practice in the majority of centers.

We present a case of DOC-producing adrenal hyperplasia with a life-threatening clinical presentation and a comprehensive review of DOC-producing adrenal lesions described in the literature. This review is intended to provide insights on the most relevant reported clinical, radiological and pathological features of DOC-producing adrenal lesions that might be useful for clinicians dealing with patients with rare causes of secondary hypertension with low or normal ALD levels.

## Methods

### Search Strategy

A systematic search was performed to identify potential relevant clinical cases in the following electronic databases: PubMed, Web of Science and EMBASE with the MeSH terms: (deoxycorticosterone OR DOC) AND (adrenal tumor OR adrenal hyperplasia). There were no language and year restrictions. All case reports published between 1965 and October 2021 were considered. The literature search was supplemented with listed references from the selected case reports and abstracts presented at scientific meetings to expand the initial search.

### Study Selection and Data Extraction

A DOC-producing adrenal lesion was defined as either an adrenal tumor or hyperplasia together with elevated DOC concentrations in peripheral and/or adrenal vein blood and/or increased DOC or tetra-hydro-DOC (THDOC) concentrations in urine, supported when available by altered enzyme immunoreactivity of the mineralocorticoid pathway and/or elevated DOC levels in adrenal tissue.

We considered the following exclusion criteria:

- Diagnosis other than adrenal carcinoma, adrenal adenoma or non-congenital adrenal hyperplasia.
- Diagnosis of adrenal carcinoma, adrenal adenoma or non-congenital adrenal hyperplasia without any major clinical sign of mineralocorticoid excess (high BP and/or hypokalemia).
- Diagnosis of adrenal carcinoma, adrenal adenoma or non-congenital adrenal hyperplasia with significant elevation of other steroid intermediates considered to be responsible for the clinical presentation of mineralocorticoid excess.
- Significant unavailable information, defined as missing data in more than four of the following eight relevant variables: sex, age at diagnosis, time of suggestive symptoms until diagnosis, overproduction of other hormones different from DOC, serum DOC levels, serum potassium levels, tumor size and histological diagnosis.

Two investigators (QA, HS) independently reviewed all articles retrieved, identified those potentially eligible for inclusion in the review and performed data extraction. In case of disagreement a third investigator was consulted (AA) and the question was solved by a consensus. Duplicated cases were excluded. A dataset was designed prior to data collection of the following items:

1. Paper meta-data: first name of author, year of publication, country of origin.
2. Baseline characteristics: gender, age at diagnosis, time of suggestive symptoms.
3. Characteristics of the DOC-producing adrenal lesion: serum potassium, ALD levels, renin levels and DOC levels, biochemical production other than DOC, tumor size and histological diagnosis.

### Quality Assessment

The present systematic review is based only on clinical cases reported in the literature of DOC-producing adrenal lesions. We included all cases found in the literature search. We carefully excluded those cases with significant missing information (more than four of the items included) to minimize any information bias. Importantly, case reports were excluded if a concomitant hormone hypersecretion considered to be more clinically relevant than DOC overproduction was present, according to the authors.

The study was registered on the Open Science Framework (https://doi.org/10.17605/OSF.IO/NR7UV) and approved by the local Ethics Committee. Written informed consent was obtained from the patient for publication of the case report.

### Hormone Measurements

Biochemical and blood parameters were analyzed using routine laboratory methods. All hormonal measurements were performed in our laboratory except 17-hydroxy-pregnenolone, DOC and 11-deoxycortisol that were measured in an external laboratory (Reference Laboratory); 11-deoxycortisol by radioimmunoassay (DIAsource ImmunoAssays, Belgium; limit of quantification (LOQ) of 0.10 ng/ml and intra and interassay coefficients of variation (CV) of 7.7 and 15.1%), and by liquid chromatography-tandem mass spectrometry (LC–MS/MS) (1290 LC-6430 QQQ Agilent; LOQ of 0.10 ng/ml, intra and interassay CV <6.7% and <8%); DOC and 17-hydroxy-pregnenolone by LC–MS/MS (1290 LC-6430 QQQ Agilent; LOQ of 1 ng/dl and 0.10 ng/ml, intra and interassay CV <6% and <6.7%, <6.8% and <8.1%, respectively).

Steroid hormones, serum testosterone and urinary cortisol (extracted with dichloromethane) concentrations were determined by an electrochemiluminescent immunoassay (cobas e601; Roche Diagnostics GmbHm, Manheim, Germany). Testosterone LOQ was 0.416 nmol/L and urinary cortisol LOQ was 0.5 nmol/L. Intra-interassay CV were <4.4% and <5.9% for testosterone and <1.1% and <1.7% for urinary cortisol. Serum estradiol and cortisol were measured by chemiluminescent microparticle immunoassay (Alinity, Abbott Laboratories, IL60064, USA). The LOQ and the intra-interassay CV were 0.088 nmol/L, <7.2% and <7.7% for serum estradiol and 27.6 nmol/L, <4.3% and <5.1% for serum cortisol. ALD and plasma renin activity (PRA) were determined by radioimmunoassay (DRG GmbH, Germany and Beckman Coulter, Immunotech Czech Republic respectively). The LOQ and the intra-interassay CV were 79 pmol/L, <11.92% and <10.15% for ALD and 0.20 µg/L/h, <11.25% and <20.9% for PRA. Progesterone, dehydroepiandrosterone sulfate (DHEAS) and androstendione were measured by chemiluminiscent immunometric assay (Immulite 1000, Siemens Healthcare Diagnostics, Llanberis, UK), with a LOQ of 1.46 nmol/L, 0.08 µmol/L and 1.0 nmol/L and intra-interassay CV of <12.5% and <13.2%, <9.5% and <15% and <9.1% and <15.2%, respectively. Serum 17-hydroxy-progesterone was measured by enzyme linked immunosorbent assay (DRG instruments GmbH, Germany) with a LOQ of 0.47 nmol/L and intra-interassay CV of <4% and <6.3%.

### Statistical Analysis

For descriptive analysis, quantitative data were expressed as mean ± SD or median (interquartile range) according to data distribution and categorical data as absolute frequencies and percentages. Distribution of continuous data was tested for normality by the Shapiro–Wilk test. Means across groups (malignant vs benign behavior) were compared using Student’s *t-*test or Wilcoxon’s rank-sum test accordingly, and Fisher’s exact test was performed to compare categorical variables. For reporting, the Preferred Reporting Items for Systematic Reviews and Meta-Analyses (PRISMA) statement (see **Supplementary Material: Tables 1, 2**) was used (5). STATA software, version 14.2 (StataCorp LLC, College Station, TX) was used for statistical analysis.

## Case Presentation

A 53-year-old Caucasian woman without any previous history of hypertension consulted the Primary Care Emergency Department for a one-week history of self-measured high BP (up to 190/100 mmHg), muscle aches and stiffness. A blood test showed serum potassium (K^+^) of 1.73 mmol/L. When reviewing previous blood tests, K^+^ was in the normal range with the exception of the immediately preceding level five months before with a result of 3.18 mmol/L. An angiotensin-converting enzyme inhibitor (enalapril 20 mg/24 h orally) and K^+^ supplements (50 KCl mEq/24 h orally) were initiated. Nevertheless, during follow-up BP was not controlled, and K^+^ levels did not improve (2.13 mmol/L). Four weeks later, she was admitted to the Intensive Care Unit (ICU) for uncontrolled hypertension (BP of 200/120 mmHg), severe hypokalemia (1.80 mmol/L), metabolic alkalosis (pH 7.52, HCO3^−^ 43.5 mmol/L) (**Table 1**) and changes in the electrocardiogram, namely, QT interval prolongation, a visible U wave and T wave flattening. In the ICU she required high doses of both intravenous and oral K^+^ (up to 176 mEq KCl/24 h). Because of initial difficulties in raising serum K^+^ levels despite high supplements, access to the bathroom was banned and a psychiatric consultation ruled out self-induced vomiting. Her BP was finally controlled with two oral drugs (the calcium channel blocker amlodipine 10 mg/24 h and the peripheral vasodilator hydralazine 25 mg/8 h) and serum K^+^ levels were maintained with in the lower limit of the normal range with oral 72 mEq/24 h of KCl.

**Table 1:** Summary of biochemistry results before (during and after the first hospitalization) and after surgery.

Laboratory test	During first hospitalization	After first hospitalization	After surgery	Reference range
**pH**	7.52	7.40	–	7.35–7.45
**pCO2 (mmHg)**	53	45.7	–	38–45
**HCO3^-^ (mmol/L)**	43.5	26	–	22–25
**Base excess (mmol/L)**	18	5.4	–	−2–2
**K^+^ (mmol/L)**	1.80	3.71	5.08	3.50–5.10
**Cl^-^ (mmol/L)**	93.4	107	–	98–107
**Na^+^ (mmol/L)**	145	143	141	136–145
**Renal function (ml/min/1.73 m^2^)**	>90	>90	>90	>90
**Urine K^+^ (mmol/L)**	28	–	–	17–83
**Urine Na^+^ (mmol/L)**	113	–	–	25–150
**Cortisol (nmol/L)**	648	–	–	171–680
**Dexamethasone suppression test (nmol/L)**	50.98	–	–	<50
**Urine cortisol (nmol/24 h)**	262.86	–	–	100–379
**ALD (pmol/L) with normal K^+^ level**	<79	81.94	696.86	187–930
**PRA (µg/L/h) with normal K^+^ level**	<0.20	<0.20	1.27	0.6-4.18
**DOC (ng/ml)**	–	35.8	19.6	2-15
**11-deoxycortisol (ng/ml)**	–	4.3 (RIA)	0.54 (LC–MS/MS)	After first hospitalization: ≤7.2
				After surgery: ≤0.79
**17-OH-pregnenolone (ng/ml)**	–	4.77	–	≤4.55
**17-OH-progesterone (nmol/L)**	–	2.99	–	<4.80
**ACTH (pmol/L)**	–	12.3	–	1.6–13.9
**LH (UI/L)**	–	33.1	–	10.4–64.6
**FSH (UI/L)**	–	50.4	–	26.7–133.4
**Estradiol (nmol/L)**	–	<0.09	–	<0.10
**Progesterone (nmol/L)**	–	1.1	–	<3.2
**Testosterone (nmol/L)**	–	0.43	–	0.22–2.90
**DHEAS (µmol/L)**	–	2.4	–	0.7–5.4
**Androstendione (nmol/L)**	–	6.3	–	3.5–11.4
**Plasma free metanephrines (nmol/L)**				
Metanephrine	–	0.10	–	<0.45
Normetanephrine	–	0.38	–	<0.74
3-metoxitiramine	–	0.04	–	<0.11
**TSH (mUI/L)**	1.84	–	1.73	0.3–5.0

pCO2, partial pressure of carbon dioxide; HCO3^-^, bicarbonate; K^+^, potassium; Cl^−^, chloride; Na^+^, sodium; ALD, aldosterone; PRA, plasma renin activity; RIA, radioimmunoassay; LC–MS/MS, liquid chromatography-tandem mass; DOC, 11-deoxycorticosterone; 17-OH-pregnenolone, 17-hydroxy-pregnenolone; 17-OH-progesterone, 17-hydroxy-progesterone; ACTH, adrenocorticotropic hormone; LH, luteinizing hormone; FSH, follicle-stimulating hormone; DHEAS, dehydroepiandrosterone sulfate; TSH, thyroid stimulating hormone.

After her hemodynamic and biochemical situation normalized, she was transferred to the hospitalization ward for diagnostic workup of suspected secondary hypertension. Her family history was irrelevant for hypertension or any other endocrine disease. Besides high BP, physical examination was normal with no signs of hypercortisolism, hirsutism or virilization. Biochemical and hormone data are shown in **Table 1**. Plasma metanephrines, 1 mg overnight dexamethasone suppression test, thyroid function and the rest of biochemical and hematological findings were normal; PRA and ALD were undetectable. Adrenal computed tomography (CT) showed two calcifications in the right adrenal gland and two adrenal nodules suggestive of adenomas in the left adrenal gland, the largest of 12.3 × 10 × 14 mm. Both nodular adrenal lesions presented −2 (the largest) and 5.4 Hounsfield Units and absolute and relative percentages of washout at 15 min of 70% and 79%, suggestive of benign lesions. Abdominal and renal ultrasonography did not reveal any abnormal finding.

At follow-up in the Endocrinology Department after hospitalization, new blood tests after adjusting antihypertensive drugs and maintaining oral K^+^ supplementation showed serum K^+^ levels in the lower normal range (3.43 mmol/L), undetectable PRA (<0.20 µg/L/h; reference range 0.6–4.18 µg/L/h) and low ALD (81.94 pmol/L; reference range 187–930 pmol/L). Further testing evidenced elevated DOC (35.8 ng/dl; reference range 2–15 ng/dl) and normality of the rest of the hormonal profile (**Table 1**). All other potential causes of non-ALD-dependent mineralocorticoid excess were excluded by clinical history, physical examination and/or biochemical results.

**Figure 1:**
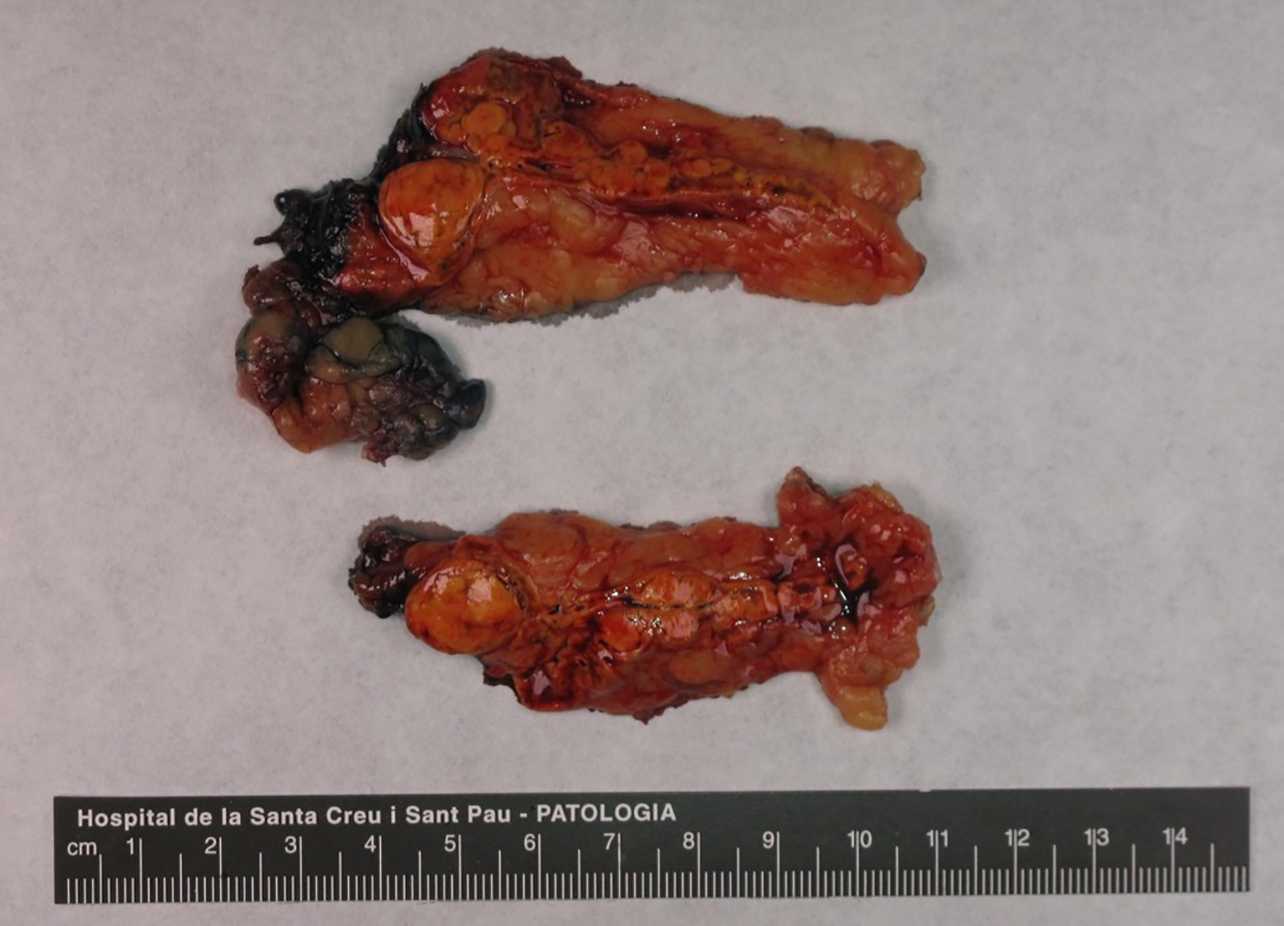
Macroscopic image of the excised adrenal gland (6 × 2 × 2 cm) with a visible multi-nodular pattern. The larger nodule was round, measured 12 mm in diameter and was composed by a bright-yellow soft tissue. Other less defined nodules were found.

**Figure 2:**
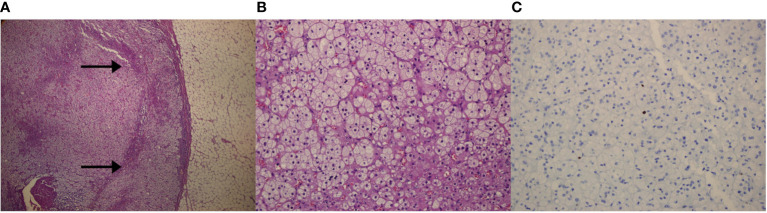
Microscopic images of the left adrenal gland. **(A)** Panoramic microscopic image of the larger nodule (Hematoxylin–Eosin; magnification ×4). Lack of capsule and fear delimitation of the normal cortex (arrows). **(B)** Representative histologic section of the adenomatous left adrenal hyperplasia, showing two types of cell population. No atypia, mitosis or necrosis was detected (Hematoxylin–Eosin; magnification ×20). **(C)** Positive Ki-67 immunohistochemical staining in less than 1% of adrenal cells (magnification ×20).

**Figure 3:**
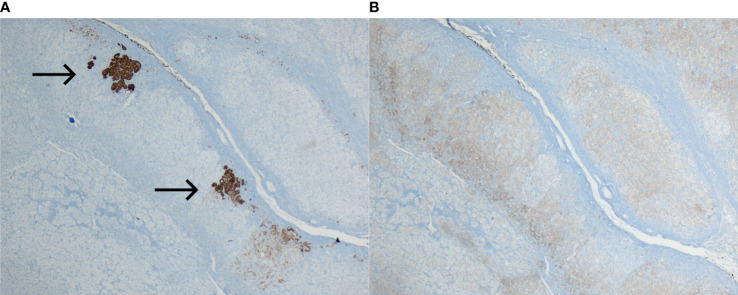
Immunohistochemical analysis of steroidogenic enzymes. **(A)** Sparse expression of CYP11B2 immunoreactivity in the zona glomerulosa. Only two clusters of ALD-producing cells stained (arrows) in the outer margin of the subcapsular zona glomerulosa (magnification ×4). **(B)** Normal expression of CYP11B1 in the zona fasciculata (magnification ×4).

**Figure 4:**
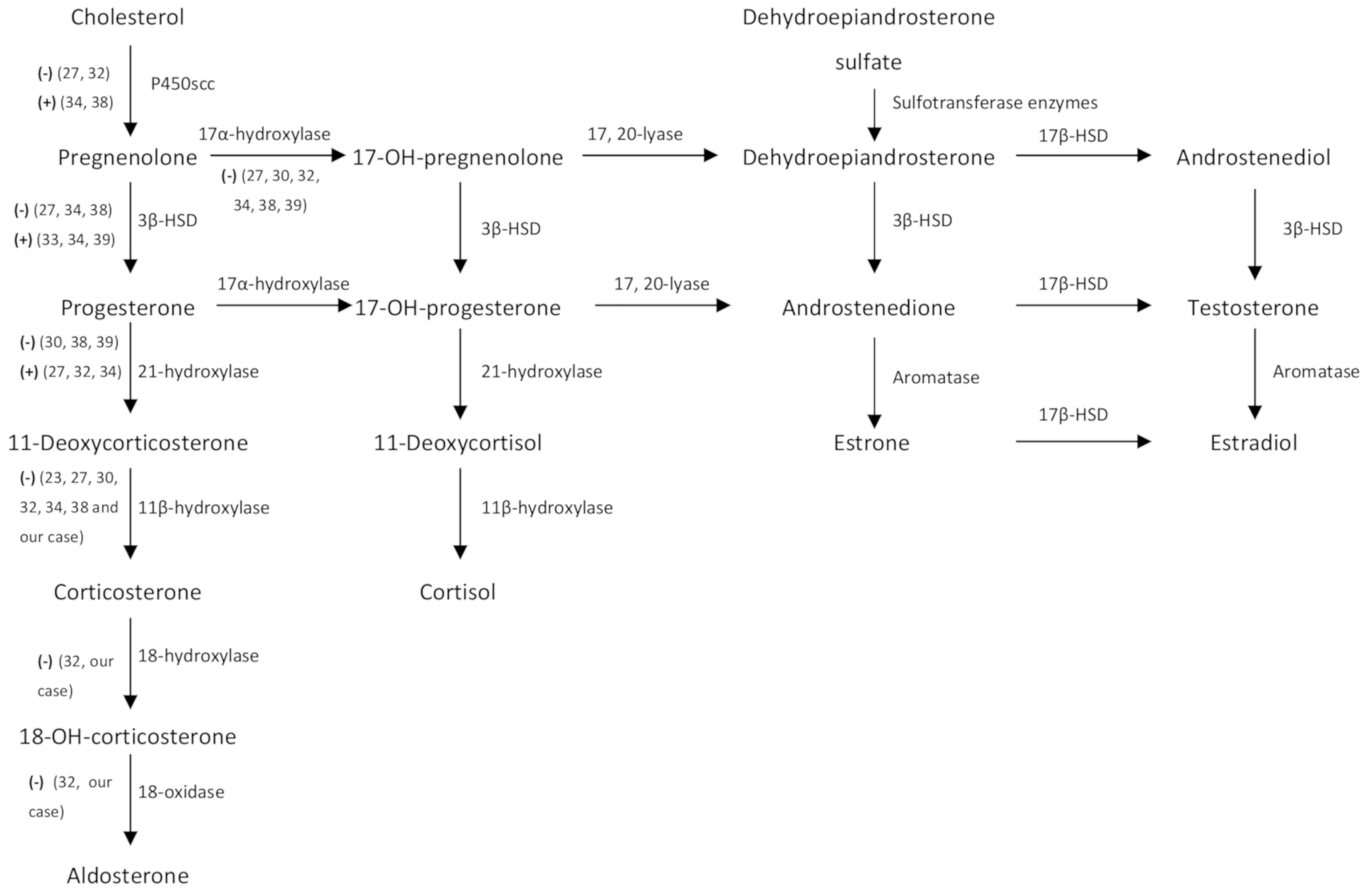
Steroid hormone metabolism pathways. (−) and (+) represent diminished and increased enzyme activity respectively detected during intratumoral analysis of steroidogenic enzymes of nine case reports (including ours). 3β-HSD, 3β-hydroxysteroid dehydrogenase; 18-OH-corticosterone, 18-hydroxy-corticosterone; 17-OH-pregnenolone, 17-hydroxy-pregnenolone; 17-OH-progesterone, 17-hydroxy-progesterone, 17β-HSD, 17β-hydroxysteroid dehydrogenase.

With the diagnostic suspicion of a functional adrenal mass due to non-ALD-dependent mineralocorticoid secretion, namely, excessive DOC production, a laparoscopic left adrenalectomy was performed. Macroscopy revealed a 6 × 2 × 2 cm left adrenal gland with cortical adenomatous hyperplasia and several soft and yellowish nodules, the largest being 12 × 12 mm and the rest were less than 1 cm and less defined (**Figure 1**). On microscopic examination, the larger nodule had no capsule and its margins were not clear (**Figure 2A**). Two main types of cells were identified: large cells disposed in irregular nests with vacuolated cytoplasm and small, round nuclei with inconspicuous nucleoli and, among these nests, a second cell population of smaller cells with eosinophilic cytoplasm (**Figure 2B**). No atypia, mitosis or necrosis was detected and the Ki67 index was positive in less than 1% of cells (**Figure 2C**). Immunohistochemical analysis of steroidogenic enzymes, namely, CYP11B2 (cytochrome P450 family 11, subfamily B, member 2) immunostaining (11β-hydroxylase, 18-hydroxylase and 18-oxidase activities) and CYP11B1 (cytochrome P450 family 11, subfamily B, member 1) immunostaining (11β-hydroxylase activity) was performed (**Figure 3**). In the zona glomerulosa of the adenomatous hyperplasia, there was a sparse expression of CYP11B2, highlighting only two isolated clusters of ALD-producing cells in the outer margin of the subcapsular area (**Figure 3A**). Immunoreactivity for CYP11B1 was fundamentally expressed in the zona fasciculata (**Figure 3B**) as expected. These findings suggested an overproduction of DOC by hyperplasic adrenal cells, leading to a suppression of ALD levels (**Figure 4**). After surgery, K^+^ levels normalized, and K^+^ supplementation could be withdrawn immediately. BP remained elevated but to a lesser degree, suggestive of co-diagnosis of primary hypertension. She required hypotensive therapy (angiotensin II receptor antagonist losartan 25 mg/24 h) at hospital discharge achieving a satisfactory BP control. Postoperative serum PRA and ALD levels were normal, while postoperative serum DOC levels practically normalized (**Table 1**).

## Results of the Systematic Review

### Study Selection

**Figure 5:**
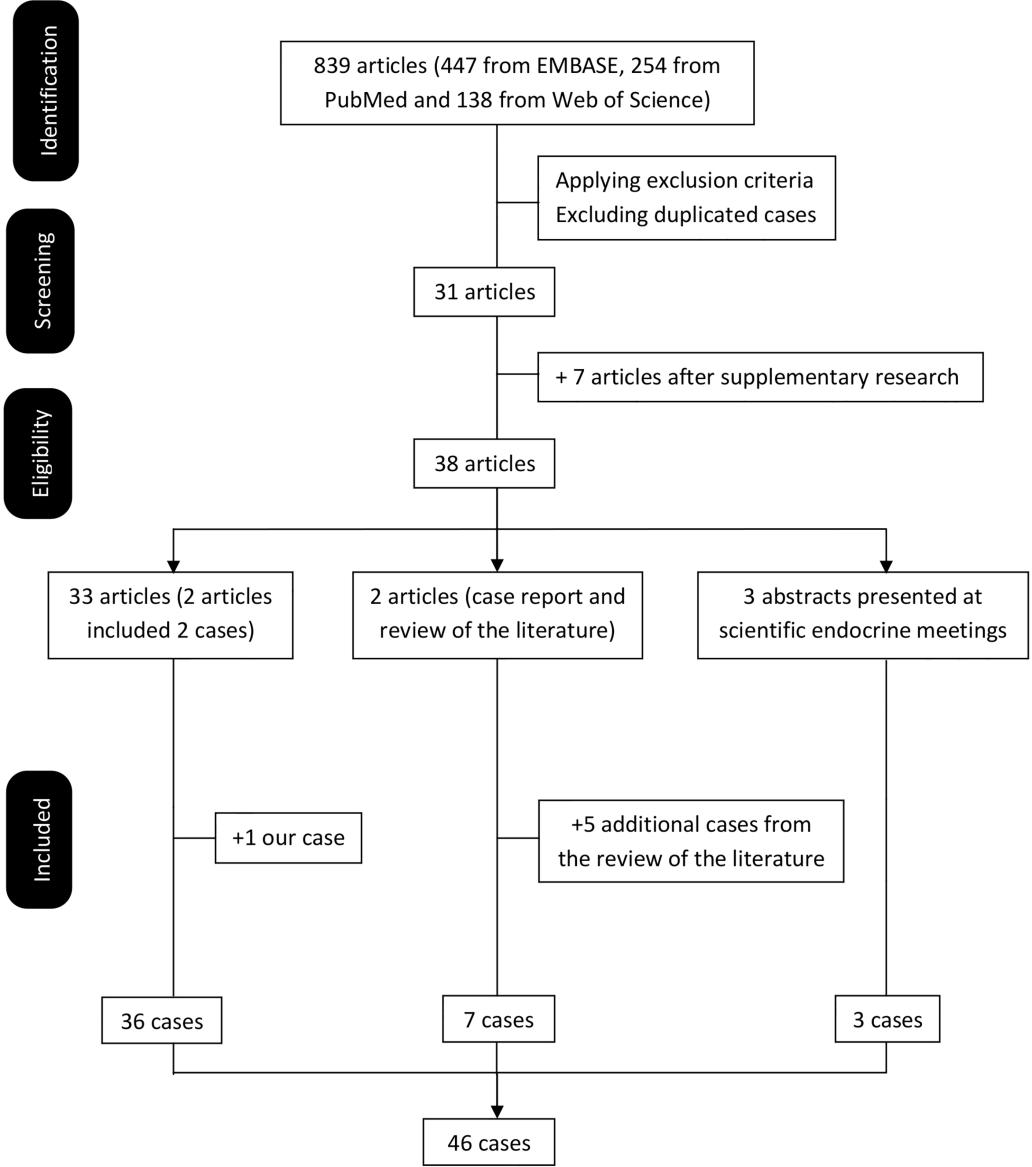
Flowchart of the literature review.

The results of the search process are summarized in a flowchart (**Figure 5** and **Supplementary Material: Table 3**). The initial search identified 839 papers. Of these 808 did not meet the eligibility criteria. Thirty-one manuscripts describing one or more case reports on DOC-adrenal producing lesions were included (6–40). Seven additional manuscripts (including one case each) were found from the listed references of the articles previously retrieved or from abstracts presented at scientific endocrine meetings (41–47). Finally, five additional cases were identified from two articles with a review of the literature in 1993 and 1995 (29). In summary, 46 cases including ours were included.

### Characteristics of DOC-Producing Adrenal Lesions

To the best of our knowledge, a total of 46 cases (including ours) of DOC overproduction due to an adrenal lesion (tumor or hyperplasia) have been reported in the medical literature. Of note, half (23, 50%) were published by Japanese authors, while the others came from the United States (11, 24.9%), United Kingdom (5, 10.9%), Spain (2, 4.3%) and France, Germany, Italy, The Netherlands and Brazil (each with 1 case, 2.2%). **Table 2** summarizes the main characteristics of the published cases of DOC overproduction by an adrenal lesion. The majority (67%) affected adult women (31 females and 15 males) with a mean age of 42.9 ± 15.2 years (range: 10.5–74 years). The most frequent clinical presentation did not differ from PA and frequently included high BP, moderate to severe hypokalemia (average of 2.68 ± 0.62 mmol/L, ranging from 1.5 to 3.8 mmol/L), and/or non-specific symptoms mostly related to hypokalemia (muscle weakness, paraesthesia). Of note, five cases presented with extremely low serum K^+^ levels <2 mmol/L (19, 33, 42, 46 and ours) and two cases did not have overt high BP (29, 41). Five cases presented with large malignant adrenal masses with abdominal (18, 22, 25, 38) or lumbar (47) compression symptoms. Median time from onset of the first suggestive symptom to date of diagnosis was 24 (55) months (range: 0 to 240 months). By definition, serum DOC concentrations were elevated in all, with wide variability from mildly to hugely elevated (from 1.5 up to 79 times above the upper limit of normal reference range). Of those reported, ALD and renin (concentration or activity) levels were mostly low or in the reference range (40/41, 98%) and decreased (30/34, 88%), respectively. On average, DOC levels were reported a median of 12.5 (18.9) times above the upper limit of normal. Overproduction of other hormones different from DOC was seen in 31 cases (67%), while only in 11 cases (24%) was DOC hormone solely produced and in 4 (9%) these data were not available.

**Table 2:** Clinical characteristics and biochemical, imaging and histological findings of DOC-producing adrenal tumors or hyperplasia. Literature review and report of a new case.

Author (year), country	Sex (F/M)	Age, years	Time of suggestive symptoms*	Serum K^+^ levels (mmol/L)**	ALD levels	Renin levels (PRA/PRC)	DOC levels***	Overproduction ≥1 adrenal hormone other than DOC	Tumor size (mm)^a^	Histological diagnosis
**Marquezy et al.** (1965)**, France** (6)	F	15	11 months	2.7	Only available in urine: <2 μg/24 h	NA	Only available in urine 10 µg/L	17-ketosteroids (DHEA), 17-hydroxycorticosteroids and estriol	NA	Carcinoma
**Biglieri (1965), USA** (41)	F	39	9 months	2.0	Only available in urine: 109 (100–205 μg/24 h)	NA	Only available in plasma from adrenal vein (0.18 µg/ml) and adrenal gland tissue (0.81 µg/g wet weight). THDOC excretion in urine 300–600 (5–30 µg/24 h)	No, however micrograms quantities of a steroid were found in the urine of the patient	NA	Bilateral gross nodular hyperplasia
**Solomon et al. (1968), USA** (7)	M	31	14 months	NA	Only available in urine 18 (60–180 µg/24 h)	NA	Only available in urine 609 (50–180 µg/24 h)	Estrogens	60–70	Carcinoma
**Powell-Jackson et al. (1974), UK** (8)	F	64	36 months	2.2	12.8 (4–18 ng/100 ml)	NA	535 (4.1–17.2 ng/100 ml)	No	94 × 77 × 58	Carcinoma
**Kondo et al. (1976)/Saruta et al. (1977)/Aiba et al. (1985), Japan** (9–11)	F	35	72 months (preeclampsia during prior pregnancy, persisting hypertension and proteinuria after delivery)	2.2	2.5 (3.0-10.0 ng/100 ml)	<0.5 (1.2–2.1 ng/ml/h)	121 (2.0–8.0 ng/100 ml)	No	95 × 85 × 55	Adenoma
**Tan et al. (1977), USA** (12)	M	10 ½	1 month	2.0	Low excretion in urine	NA	1,560 (10–180 pg/ml)	17-OH-progesterone, 11-deoxycortisol and cortisol	NA	Carcinoma
**Hogan et al. (1977), USA** (13)	M	45	12 months	3.7 on supplements	14.2 (2–14 ng/dl)	0.75 (2–12 ng/ml/3h)	126.1 (5–15 ng/dl)	Corticosterone, tetrahydrocorticosterone, tetrahydro-11-deoxycortisol and cortisol	50	Adenoma
**Davies et al. (1980)/Kelly et al. (1982), UK** (14, 15)	F	51	18 months	2.9	192 (100–500 pmol/L)	<0.5 (1–6 ng/ml/h)	12,400 (100–500 pmol/L)	Progesterone	NA	Carcinoma
**Kojima (1984), Japan** (29)	M	56	NA	NA	NA		NA	NA	15 × 15 × 30	Adenoma
**Honjo (1987)/Miki (1988), Japan** (29)	M	44	NA	NA	NA		NA	NA	180 × 155 × 120	Carcinoma
**Isles et al. (1987), UK** (16)	F	47	Several years	2.8	800 (<500 pmol/L)	<1 (9–50 µU/L)	5,117 (120–480 pmol/L)	Corticosterone, 18-OH-corticosterone and ALD	56 × 30 × 36	Carcinoma
**Makino et al. (1987), Japan** (17)	F	25	60 months	3.7	12.0 (5.6–16.4 ng/dl)	0.1 (0.3–3.2 ng/ml/h)	244 (1–30 ng/dl)	Pregnenolone, progesterone, 18-OH-DOC, corticosterone, 18-OH-corticosterone, 17-OH-pregnenolone and 17-OH-progesterone	35 × 30 × 35	Carcinoma
**Irony et al. (1987), USA** (18)	F	31	60 months	2.3	<83 (205 ± 25 pmol/L)	0.03 (0.61 ± 0.08 ng/L·s)	5,659 (148–178 pmol/L)	18-OH-DOC and corticosterone	60	Adenoma
	F	55	1 month	2.8	144 (205 ± 25 pmol/L)	0.11 (0.61 ± 0.08 ng/L·s)	14,010 (148–178 pmol/L)	18-OH-DOC, corticosterone and 18-OH-corticosterone	NA	Carcinoma (biopsy)
**Ishikawa et al. (1988), Japan** (19)	F	52	48 months	2.2	0.12 (0.03–0.18 nmol/L)	0.08 (0.3–2.9 ng/ml/h)	1.13 (0.06–0.23 nmol/L)	Corticosterone	15	Adenoma
	F	62	24 months	1.7	0.04 (0.03–0.18 nmol/L)	0.10 (0.3–2.9 ng/ml/h)	1.47 (0.06–0.23 nmol/L)	Corticosterone and 11-deoxycortisol	CT scan did not show an adrenal tumor	Adrenal hyperplasia?
**Konta et al. (1989)/Osanai et al. (1990), Japan** (20, 21)	F	33	84 months	2.1	60 (50–360 pmol/L)	0.1 ng/ml/h	9.68 (0.034–0.325 ng/ml)	11-deoxycortisol	21 × 15	Adenoma
**Sakurai et al. (1989), Japan** (22)	M	40	60 months	3.8	65 (10.9–62.7 pg/ml)	0.2 (0.3–2.9 ng/ml/h)	3.360 (0.08–0.278 ng/ml)	Pregnenolone, progesterone and ALD	90 × 70 × 50	Carcinoma
**Road/Okano (1989), Japan** (29)	M	28	NA	3.1	NA		NA	NA	68 × 73 × 50	Carcinoma
**Imada/Terayama/Komatsu (1989–1990), Japan** (29)	F	33	NA	3.1	NA		NA	NA	21 × 15 × 11	Adenoma
**Saha et al. (1990), Japan** (23)	F	39	NA	3.0	3 (3–10 ng/dl)	0 (1.2–2.1 ng/ml/h)	1.16 (0.03–0.33 ng/ml)	No	48	Adenoma
**Yoshitomi (1990), Japan** (29)	F	74	NA	NA	NA		NA	No	40	NA
**White et al. (1992), USA** (24)	F	43	48 months	3.0	444 (83–694 pmol/L)	0.03 (0.08–0.53 ng/L/s)	7,260 (61–580 pmol/L)	Progesterone, 18-OH-DOC, corticosterone and 11-deoxycortisol	50	Adenoma
**Bijl et al. (1992), Netherlands** (25)	M	73	0 month (hospital admission)	2.8	0.19 (0.14–0.22 nmol/L)	0.30 (0.30–0.60 nmol/L)	9.5 (<3.0 nmol/L)	Progesterone and 17-OH-progesterone	200	Carcinoma
**Matsumoto et al. (1993), Japan** (26)	M	43	36 months	NA	Low in plasma and in urine <2 (2–14 ng/dl)	0.6 (0.3–3.0 ng/ml/h)	0.26 (<0.13 ng/ml)	18-OH-DOC, corticosterone and 18- OH-corticosterone	30 × 30^†^	Adenoma
**Yamamoto, et al. (1993), Japan** (27)	M	66	4 months	2.6	47 (35.7–240 pg/ml)	Suppressed (<0.1 ng/ml/h)	17.10 (0.08–0.278 ng/ml)	Progesterone and 18-OH- DOC	100 × 80 × 45	Carcinoma
**Komura et al. (1993), Japan** (28)	F	55	Several months	2.8	98 (37.5–240 pg/ml)	0.2 (0.3–2.9 ng/ml/h)	1.0 (<0.130 ng/ml)	18-OH-DOC	40 × 20^†^	Adenoma
**Furuse et al. (1995), Japan** (29)	M	39	0 month (hospital admission)	3.2	6.5 (2–12 ng/dl)	NA	4.86 (0.08-0.28 ng/ml)	Pregnenolone, progesterone, 11-deoxycortisol and androstenedione	110 × 60 × 50	Carcinoma
**Wada et al. (1995), Japan** (30)	F	29	3 months	2.5	45.2 (50–150 pg/ml)	0.01 (0.2–2.0 ng/ml/h)	11.6 (0.03–0.33 ng/ml)	18-OH-DOC, corticosterone, 11-deoxycortisol and cortisone	28 × 44^†^	Adenoma
**Nitta et al. (1996), Japan** (31)	M	58	84 months	3.0	98.2 (47–131 ng/ml)	0.33 (0.5–2.0 ng/ml/h)	1.69 (0.08–0.278 ng/ml)	Corticosterone	Right adrenal tumor: 22 × 20 Left adrenal tumor:10 × 10	Bilateral adenoma
**Toyoda et al. (1996), Japan** (32)	F	55	120 months	2.5	98 (37.5–240 pg/ml)	0.2 (0.3–2.9 ng/ml/h)	2.82 (<0.13 ng/ml)	18-OH-DOC	40	Adenoma
**Limone et al. (1997), Italy** (33)	F	23	6 months	3.6	Only available in urine (normal)	NA	NA in serum. THDOC excretion in urine 78 (25–60 µg/24 h)	No	NA (micro-adenomas)	Micronodular hyperplasia
**Egoshi et al. (1998), Japan** (34)	F	60	240 months	1.6	Normal		6.64 (<0.3 ng/ml)	11-deoxycortisol	110 × 85 × 45	Carcinoma
**Soranno et al. (1999), USA** (35)	F	14	36 months	3.4	<30 (50–250 pmoL/L)	0.15 (0.20–1.60 ng/ml/h)	5.7 (0.05–0.26 nmol/L)	17-OH-pregnenolone, 17-OH-progesterone, 11-deoxycortisol, DHEA, DHEAS, A4-androstenedione and testosterone	50	Adenoma
**Nagai et al. (1999), Japan** (42)	F	31	11 months	1.9	62 (35.7–240 pg/ml)	0.1 (0.3–2.9 ng/ml/h)	1.68 (0.03–0.33 ng/ml)	No	11 × 9 × 8	Adenoma
**Müssig et al. (2005), Germany** (36)	M	37	Some weeks	3.4	43 (10–160 pg/ml)	0.2 (0.12–1.59 ng/ml/h)	347.2 (2–15 ng/dl)	17-OH-pregnenolone and 11-deoxycortisol	140 × 90 × 70	Carcinoma
**Messer et al. (2007), USA** (37)	F	42	5 months	2.3	<1 (1–16 ng/dl)	2.35 (4.0–7.7 ng/ml/h)	385 (2–19 ng/dl)	17-OH-progesterone, cortisol, androstenedione, testosterone and estradiol	80 × 55 × 35	Carcinoma
**Sone et al. (2009), Japan** (38)	F	27	0 month (hospital admission)	2.3	66 (30–159 pg/ml)	0.1 (0.2–2.7 ng/ml/h)	8.04 (0.03–0.33 ng/ml)	Pregnenolone, progesterone 18-OH-DOC and 17-OH-progesterone	170 × 110 × 78	Carcinoma
**Gupta et al. (2010), USA** (43)	F	35	216 months	3.1	Suppressed (<4.0 ng/dl)	<0.6 (0.6–4.0 ng/ml/h)	65 (1.6–5.6 ng/dl)	Cortisol	53 × 45 × 70^†^	Adenoma
**Freel et al. (2014), UK** (44)	M	42	NA	2.3	Suppressed		NA	Corticosterone and 11-deoxycortisol	120 × 80	Carcinoma
**Oyama et al. (2014), Japan** (39)	M	59	120 months	3.0	154 (29.9–159 pg/ml)	0.2 (0.3–2.9 ng/ml/h)	1.52 (0.08–0.28 µg/ml)	ALD	25 × 25	Macroadenoma producing DOC and microadenomas producing ALD
**Paja et al. (2017), Spain** (45)	F	29	12 months	3.1	Normal to low (7.3 ng/dl)	Suppressed (<0.2 ng/ml/h)	237.9 (<15 ng/dl)	No	65	Adenoma
**Goodale et al. (2018), USA** (46)	F	53	60 months	1.5	4 (4–31 ng/dl)	2.0 (0.5–4.0 ng/ml/h)	62.60 (<19 ng/dl)	No	63 × 34 × 52^†^	Carcinoma
**Marques et al. (2019), UK** (40)	F	35	120 months	Long standing hypokalemia 3.7 on supplements	224 (330–830 pmol/L)	0.01 (0.15–2.10 pmol/ml/h)	9,741 (121–514 pmol/L)	No	110 × 75 × 60	Neoplasm of uncertain malignant potential
**Scaranello et al. (2020), Brazil** (47)	F	61	36 months	2.4	Suppressed		654 (<25 ng/dl)	Progesterone and 11- deoxycortisol	240 × 130 × 130	Carcinoma
**The current case reported (2022), Spain**	F	53	1 month	1.7	<79 (187–930 pmol/L)	<0.2 (0.6–4.18 μg/L/h)	35.8 (2–15 ng/ml)	No	The biggest adrenal nodule 12 × 12	Unilateral adenomatous hyperplasia
**Summary/Total#**	**31 F (67%), 15 M (33%)**	**42.9 ± 15.2**	**24** (55)	**2.68 ± 0.62**	**25 in the reference range (54.3%) 15 decreased (32.6%) 1 elevated (2.2%) 5 not reported (10.9%)**	**30 decreased (65.2%) 4 in range (8.7%) 12 not reported (26.1%)**	**12.5 (18.9)**	**31 yes (67%), 11 no (24%), 4 not reported (9%)**	**61.5** (60)	**21 (45.7%) ACC 19 (41.3%) benign 4 (8.7%) hyperplasia 1 (2.2%) uncertain malignant potential 1 (2.2%) not reported**

*The estimation has been made considering the onset of symptoms (hypertension and/or symptoms of hypokalemia) until the patient was referred or admitted to a medical centre. ** The minimum K^+^ level is reported. *** Median (IQR) times above the upper limit of normal range of serum DOC levels reported in each article and in parentheses the normal reference values.

^a^Average tumor size was calculated based on the maximum diameter reported. ^†^Size information obtained from CT scan and/or MRI. #Quantitative data are expressed as the mean SD (Gaussian distribution) or median (interquartile range) (non-Gaussian distribution) and categorical data as absolute frequencies and percentages. F, female; M, male; K^+^, potassium; ALD, aldosterone; PRA, plasma renin activity; PRC, plasma renin concentration; DOC, 11-deoxycorticosterone; NA, not available; DHEA, dehydroepiandrosterone; THDOC, tetra-hydro-11- deoxycorticosterone; 17-OH-progesterone, 17-hydroxy-progesterone; 18-OH-corticosterone, 18-hydroxy-corticosterone; 18-OH-DOC, 18-hydroxy-11-deoxycorticosterone; 17-OH-pregnenolone, 17-hydroxy-pregnenolone; CT, computed tomography; DHEAS, dehydroepiandrosterone sulphate; ACC, adrenocortical carcinoma; SD, standard deviation; MRI, magnetic resonance imaging.

The most common histological types were carcinoma (21, 45.7%) and adenoma (19, 41.3%). Other less frequent pathological types when reported were adrenal hyperplasia (4, 8.9%) and a neoplasm of uncertain malignant potential (2.2%) and no available information in one case (2.2%). Excluding DOC-producing adrenal hyperplasia, median calculated tumor size based on the maximum diameter reported was 61.5 (60) mm (range: 11–240 mm). When comparing by histological behavior, malignant compared to benign lesions were larger (tumor size of 97, (70) vs 40 (28) mm, p = 0.0001), with higher DOC levels (20.3 (14.1) vs 7.4 (17.8) times above the upper limit of normal, p = 0.036) and a shorter time of evolution of symptoms (11 (35) vs 42 (74) months, p = 0.029). Malignant lesions in comparison with benign lesions were not significantly different in gender distribution (malignant: men 10 (45%)/women 12 (55%) vs benign: men 5 (22%)/women 18 (78%), p = 0.092), K^+^ levels (2.69 ± 0.14 vs 2.67 ± 0.14 mmol/L, p = 0.936) or existence of concomitant hormone overproduction (17 (85%) vs 14 (67%), p = 0.172).

## Discussion

To our knowledge, this is the forty-sixth DOC overproduction due to an adrenal lesion published so far worldwide. This case is remarkable for the rapid and severe clinical presentation, despite the relatively small size of the adenomatous adrenal hyperplasia (the largest nodule of 12 mm) and mildly elevated DOC levels compared to the majority of cases reported. Our literature review revealed a female predominance, average age of presentation in the fourth decade, a median tumor size of around 62 mm and a benign behavior in roughly half of the DOC-producing adrenal lesions (including adrenal adenomas and hyperplasia). This review adds knowledge in the limited body of literature on this extremely rare functional adrenocortical lesion and is intended to raise awareness of the possibility of a DOC-producing adrenal lesion in the presence of low serum ALD levels despite hypertension presumed to be due to excessive mineralocorticoid secretion.

Mineralocorticoids are, together with glucocorticoids and sex steroids, the three different adrenal steroid hormones produced in the adrenal cortex (**Figure 4**). ALD is the final and most potent mineralocorticoid hormone, representing 90% of mineralocorticoid activity (48). However, other mineralocorticoid products have been isolated from the adrenal cortex (48). These are usually secreted in small amounts and have a weaker mineralocorticoid activity (48). Thus, it seems plausible that only high levels of mineralocorticoid precursors different from ALD could produce a clinically relevant hypermineralocorticoid state.

**Figure 6:**
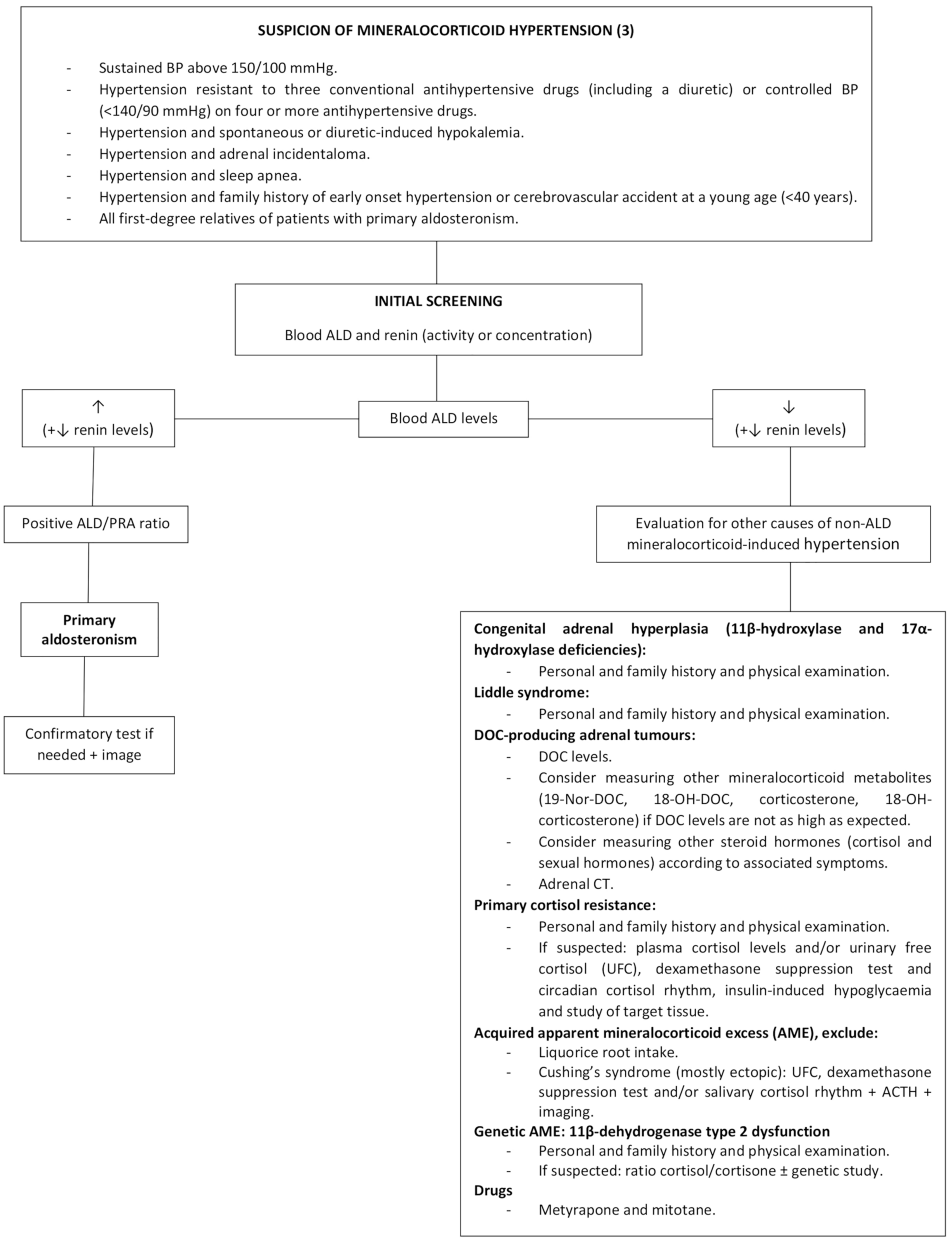
Algorithm for the diagnostic approach of patients with non-ALD-dependent mineralocorticoid hypertension. BP, blood pressure; ALD, aldosterone; PRA, plasma renin activity; DOC, 11-deoxycorticosterone; 19-Nor-DOC, 19-Nor-deoxycorticosterone; 18-OH-DOC, 18-hydroxy-deoxycorticosterone; CT, computed tomography; ACTH, adrenocorticotropic hormone.

DOC is an ALD precursor with a 1/20 (38) to 1/30 (48) of the mineralocorticoid activity of ALD. It is synthesized from progesterone by the 21α-hydroxylase enzyme in the adrenal cortex and converted to corticosterone by the 11β-hydroxylase enzyme (**Figure 4**). Four uncommon situations generate DOC excess with low ARP and ALD levels: congenital adrenal hyperplasia (CAH) due to 11β-hydroxylase and 17α-hydroxylase deficiency, DOC-producing adrenal tumors, primary cortisol resistance and apparent mineralocorticoid excess (AME) syndrome due to acquired or genetic 11β-dehydrogenase type 2 dysfunction. An algorithm for the differential diagnosis of non-ALD-dependent mineralocorticoid hypertension (also known as pseudohyperaldosteronism) is shown in **Figure 6**. An accurate diagnosis is crucial since appropriate treatment differs substantially among each clinical entity. In our case, CAH was rapidly ruled out since the patient did not have any sign or symptom of hirsutism or virilization, nor relevant family history or consanguinity and the 5th decade is not a common age of presentation for CAH. Genetic AME syndrome, an autosomal recessive inherited disease usually presents in childhood, was excluded and also acquired causes (i.e., liquorice root intake and Cushing’s syndrome). Moreover, DOC concentrations might increase in patients receiving 11 β-hydroxylase inhibitors such as metyrapone or mitotane, which was not the case. Furthermore, the CT scan revealed an adrenal mass, increasing suspicion of a DOC-producing adrenal lesion.

The first evidence of adrenal tumor DOC production emerged in the sixties (49, 50) and was published in 1965 by Marquezy et al. and Biglieri (6, 41). Overproduction of DOC was determined by an excess of DOC and its metabolite THDOC excreted in 24 h urine (6, 41) and by an increased content of DOC in adrenal tissue and adrenal venous plasma (41). Two main factors can mask the diagnosis and contribute to underestimating its incidence. On the one hand, DOC assay is not readily available in many routine clinical centers (40). On the other hand, some cases of PA might occur simultaneously with DOC overproduction (16, 39), implying that DOC is not searched for since ALD is not suppressed.

Our case report shows several singularities. One was the suddenness of the life-threatening clinical presentation that took the patient to the ICU in less than a month since high BP was detected, while most of the previously published cases presented months or years before diagnosis (16, 20, 31, 32, 34, 39, 40, 43). Additionally, initial serum hypokalemia was resistant despite high intravenous and oral K^+^ supplements, leading to the suspicion of an underlying psychiatric disorder despite not having any previous history of mental diseases. The combination of severe hypokalemia, metabolic alkalosis, critical hypertension and the presence of two left adrenal adenomas were highly suggestive of mineralocorticoid hypertension, yet ARP and ALD levels were repeatedly very low, excluding PA, while elevated serum DOC led to the diagnosis of a DOC-producing adrenal neoplasm (**Figure 6**).

As reported, very high DOC plasma levels are to be expected, the highest published up to eighty times above the upper normal limit (18). However, in our case, DOC levels only doubled the normal range. Similarly, Matsumoto et al. (26) reported a case of weak mineralocorticoid-producing benign adrenal tumor with a doubling of serum DOC levels. The authors hypothesized that another mineralocorticoid intermediate different from DOC, 18-hydroxy-deoxycorticosterone (18-OH-DOC, a hydroxylated metabolite of DOC), that was above the normal value, might be responsible for the hypertensive state of that patient (26). In addition to 18-OH-DOC, other mineralocorticoid precursor related to DOC, such as 19-Nor-DOC, can cause overt hypertension (51), especially in patients with slightly elevated DOC levels. Unfortunately, we could not measure 18-OH-DOC or 19-Nor-DOC. Although in the presented patient postoperative hormone levels almost normalized, supporting the indubitable participation of DOC overproduction, it is possible that other metabolites of DOC with a known hypertensive and salt-retaining activity were also involved.

Regarding histological behavior, malignant DOC-producing adrenal lesions were larger (about 2.5 times more) and had higher DOC levels (about 3 times higher) in comparison to benign lesions; without differences in gender distribution, age and K^+^ levels. Thus, the benign behavior of our case with a relatively small tumor size and mildly elevated DOC levels could be expected based on the review of DOC-producing adrenal lesions published so far.

First physiopathological reports on the mechanisms involved in DOC-producing adrenal tumors showed a relative 11β-hydroxylation deficiency in an adrenal carcinoma tissue of a deceased patient (50). Since then, intratumoral analysis of steroidogenic enzymes (**Figure 4** **)** revealed a reduced 11β-hydroxylase and 17α-hydroxylase enzyme activities in all instances (23, 27, 30, 32, 34, 38, 39 and our case), suggestive of an autonomous DOC overproduction by adrenal neoplastic cells.

Recently, LC–MS/MS metabolite steroid profiling in urine followed by machine learning analysis have evidenced a panel of nine steroids as a highly discriminative tool in differentiating adrenocortical carcinoma from adenoma (52), being tetra-hydro-11-deoxycortisol (a metabolite of 11-deoxycortisol) the most distinguishable biomarker. This panel also included THDOC, a metabolite of DOC, which was significantly increased in patients with adrenocortical carcinoma (52). Similarly, urine metabolite steroid profiling of DOC-producing adrenal tumors could permit to better differentiate the behavior of these lesions.

Some limitations need to be considered. Unfortunately, we were not able to measure DOC intermediate products. However, the PA-like clinical presentation, the biochemical and hormonal profile at diagnosis, the immunohistochemical analysis in the surgical specimen together with the marked clinical, biochemical and hormonal improvement after surgery, led to confirm the diagnosis of DOC overproduction due to unilateral adenomatous adrenal hyperplasia. We might have not identified all cases reported and cannot rule out the possibility that some unpublished cases of interest may have been missed. Nevertheless, we collected an important number of cases of this very rare disease to provide enough accurate data to summarize its main characteristics and increase the knowledge of DOC-producing adrenal lesions. Besides, we had limited access to case reports written in Japanese, but all cases found were computed and the scant information available was included.

In conclusion, DOC overproduction due to adrenal lesions mostly affects middle aged women with a PA-like presentation and time to diagnosis of several months. Nearly half of the cases demonstrated malignant behavior and adrenal hyperplasia was exceptional. Reduced 11β-hydroxylase and 17α-hydroxylase enzyme activities were the most frequent immunohistochemical findings.

## Data Availability Statement

The raw data supporting the conclusions of this article will be made available by the authors, without undue reservation.

## Author Contributions

AA planned the concept of this review. QA and HS carried out the literature research, manuscript selection and data extraction. AA performed the analysis. QA and AA drafted the manuscript. EU performed the laboratory analysis. EL provided the macroscopic and microscopic images and MR performed the immunohistochemical analysis. All authors listed have made a substantial, direct, and intellectual contribution to the work and approved it for publication.

## Conflict of Interest

The authors declare that the research was conducted in the absence of any commercial or financial relationships that could be construed as a potential conflict of interest.

## Publisher’s Note

All claims expressed in this article are solely those of the authors and do not necessarily represent those of their affiliated organizations, or those of the publisher, the editors and the reviewers. Any product that may be evaluated in this article, or claim that may be made by its manufacturer, is not guaranteed or endorsed by the publisher.
