# Supplementary material for: 11-Deoxycorticosterone Producing Adrenal Hyperplasia as a Very Unusual Cause of Endocrine Hypertension: Case Report and Systematic Review of the Literature
Source: Front Endocrinol (Lausanne). 2022 Mar 31;13:846865. doi: 10.3389/fendo.2022.846865 (PMC9008131; doi:10.3389/fendo.2022.846865)
Supplement: Supplementary file 3 [file Table_3.docx]

**Identification of studies via other methods**

**Identification of studies via databases and registers**

Records identified from:

Citation searching (n =7)

Records removed *before screening*:

Duplicate records removed (n =41)

Records marked as ineligible by automation tools (n =0)

Records removed for other reasons (n =0)

Records identified from*:

Databases (n =839)

Registers (n =0)

**Identification**

Records screened

(n =798)

Records excluded**

(n =765)

Reports not retrieved

(n =0)

Reports sought for retrieval

(n =7)

Reports sought for retrieval

(n =33)

Reports not retrieved

(n =2)

**Screening**

Reports assessed for eligibility

(n =7)

Reports excluded:0

Reports assessed for eligibility

(n =31)

Reports excluded:0

Studies included in review

(n =38)

**Included**

*Consider, if feasible to do so, reporting the number of records identified from each database or register searched (rather than the total number across all databases/registers).

**If automation tools were used, indicate how many records were excluded by a human and how many were excluded by automation tools.

*From:*  Page MJ, McKenzie JE, Bossuyt PM, Boutron I, Hoffmann TC, Mulrow CD, et al. The PRISMA 2020 statement: an updated guideline for reporting systematic reviews. BMJ 2021;372:n71. doi: 10.1136/bmj.n71. For more information, visit:<http://www.prisma-statement.org/>
